# Efficacy and tolerability of a new formulation of artesunate-mefloquine for the treatment of uncomplicated malaria in adult in Senegal: open randomized trial

**DOI:** 10.1186/1475-2875-11-416

**Published:** 2012-12-12

**Authors:** Roger CK Tine, Babacar Faye, Khadime Sylla, Jean L Ndiaye, Magatte Ndiaye, Doudou Sow, Aminata C Lo, Annie Abiola, Mamadou C Ba, Oumar Gaye

**Affiliations:** 1 Department of Parasitology, Faculty of Medicine, University Cheikh Anta DIOP, Dakar, Senegal

## Abstract

**Background:**

Prompt treatment of malaria attacks with arteminisin-based combination therapy (ACT) is an essential tool for malaria control. A new co-blister tablet of artesunate-mefloquine (AM) with 25 mg/kg mefloquine has been developed for the management of uncomplicated malaria attacks. This non-inferiority randomized trial, was conducted to evaluate the efficacy and safety of the new formulation of AM in comparison to artemether-lumefantrine (AL) for the treatment of acute uncomplicated *Plasmodium falciparum* malaria in adults in Senegal.

**Methods:**

The study was carried out from September to December 2010 in two health centres in Senegal. The study end points included (i) PCR corrected adequate clinical and parasitological response (ACPR) at day 28, (ii) ACPR at days 42 and 63, (iii) parasites and fever clearance time, (iv) incidence of adverse events and patients biological profile at day 7 using the WHO 2003 protocol for anti-malarial drug evaluation.

**Results:**

Overall, 310 patients were randomized to receive either AM (n = 157) or AL (n = 153). PCR corrected ACPR at day 28 was at 95.5% in the AM arm while that in the AL arm was at 96.7% (p = 0.83). Therapeutic efficacy was at 98.5% in the AM arm versus 98.2% in the AL group at day 42 (p = 1). At day 63, ACPR in the AM and AL arms was at 98.2% and 97.7%, respectively (p = 0.32). The two treatments were well tolerated with similar biological profile at day 7. However, dizziness was more frequent in the AM arm.

**Conclusion:**

Artesunate-mefloquine (25 mg/Kg mefloquine) is efficacious and well-tolerated for the treatment of uncomplicated *P. falciparum* malaria in adult patients.

## Background

Malaria remains a major public health problem in tropical regions. According to the World Health Organization (WHO), in 2010 there were an estimated 149 to 274 million cases and 537,000 to 907,000 deaths worldwide [1]. Over 86% of these deaths occur in children under five years of age [1]. The development and spread of multidrug-resistant *Plasmodium falciparum*, particularly for the previous main anti-malarial drugs, such as chloroquine and sulphadoxine-pyrimethamine, has affected efforts to control malaria, and can lead to an increase in malaria mortality and morbidity if ineffective drugs remain the standard of care after drug-resistant strains become established. To deal with the threat of resistance of *P. falciparum* to monotherapies, combinations of anti-malarial drugs are now recommended by the WHO.

Artemisinin-based combination therapy (ACT) is widely promoted as a strategy to counteract the increasing resistance of *P. falciparum* to anti-malarial drugs, prevent disease transmission and reduce the risk of drug resistance [2-4]. ACT can rapidly reduce the parasite biomass, and may also prevent transmission of *P. falciparum* by acting against gametocytes [5,6]. Several formulations of ACT are currently available for the treatment of uncomplicated malaria. A co-blister tablet formulation of artesunate-mefloquine (Artequin®) has been developed by Mepha ^Ltd^ and was initially used on the basis of 750 mg mefloquine, which represents 15 mg/kg mefloquine per treatment. Studies in Asian countries showed that artesunate-mefloquine (AM) is effective and safe for the treatment of uncomplicated malaria [7]. In Africa, several studies demonstrated its efficacy and tolerability [8,9]. However, the dosage of 15 mg/kg mefloquine base is below the posology recommended by the WHO (25 mg/kg mefloquine), to avoid resistance to mefloquine. As a line extension of the co-blister tablet, a co-formulation of AM was developed for the treatment of paediatric patients. Following a recent change in recommendations of WHO for areas of high malaria transmission, the total mefloquine dosage in the co-blister tablet formulation and the novel paediatric formulation was increased to 25 mg/kg body weight [2]. Although the paediatric co-formulation was safe and effective in children less than 5 years [10], studies aiming at evaluating the new co-blister in adults patients have become relevant in the context of scaling up anti-malarial drugs in African countries, in order to continue to monitor adverse drug reactions as well as drug efficacy. The aim of this study was to compare the clinical and parasitological efficacy, as well as the clinical safety, laboratory profile, of a new formulation of artesunate-mefloquine (AM) (25 mg/kg mefloquine base) compared to artemether-lumefantrine (AL) for the treatment of uncomplicated *P. falciparum* malaria in adults in Senegal.

## Methods

### Study area and population

The study was carried out from September to December 2010 in the two health centres of Guediawaye (20 km from Dakar, the capital city), and Kaolack, 200 km from Dakar (Senegal). Malaria transmission in the two sites is highly seasonal with a transmission peak from September to December. *Plasmodium falciparum* is the predominant parasite species and transmission is mainly due to *Anopheles gambiae s.l.* (Konate *et al.* unpublished data).

Adult patients with acute uncomplicated *P. falciparum* malaria seen at study sites with parasite density ranging from 1,000 to 100,000 parasites/μL, and presence of an axillary temperature >37.5°C or history of fever during the previous 24 hours, were included in the study. Patients were excluded if they had signs of severe malaria such as convulsions, severe anaemia, respiratory distress, jaundice, haemoglobinuria or/and had a history of allergy to the study drugs. Informed consent was required prior to each study participant enrolment.

### Study design

The study was designed as an open randomized, non-inferiority trial with two parallel groups, comparing three-day regimens of AM versus AL. The primary end point was represented by the PCR corrected adequate clinical and parasitological response at day 28. Secondary end points were represented by (i) adequate clinical and parasitological response at days 42 and 63, (ii) parasites and fever clearance time, (iii) incidence of adverse events in the two groups, (iv) patients’ biological profile at day 7 in the two groups.

### Treatment

Patients in the AM arm received one tablet of artesunate (200 mg) and one tablet of mefloquine (500 mg) everyday for three days, representing a total dose of 600 mg of artesunate and 1,500 mg of mefloquine. In the AL arm, patients received four tablets twice daily for three days. Each tablet of AL contains 20 mg of lumefantrine and 120 mg of artemether. Treatment doses were administered at health centres under the supervision of the study physician. In the event of a patient vomiting within 30 minutes of tablet intake, the dose was repeated. In the event of a second vomiting episode, the patient was withdrawn and received quinine in accordance with the national malaria control programme guidelines.

### Data collection

The WHO 2003 protocol for anti-malarial drug efficacy evaluation was used in this study [11]. After inclusion, patients were evaluated every day during treatment (days 0, 1, 2), on day 3 and at follow up on days 7,14,21, 28. In a subgroup of randomly selected study participants (representing 30% of the total sample), the follow up was extended at days 42 and 63 to assess the long term protective effect of each drug after curative doses. During each scheduled visit, patients were interviewed and examined by the study physician to assess their clinical conditions as well as adverse events. All signs noted during the interview and clinical examinations were reported on a case reported form (CRF). Thick and thin smears were performed at each scheduled visit; four drops of blood were collected on filter paper at Day 0 and at day of recurrent parasitaemia for genotyping in order to distinguish recrudescence from new infection.

### Laboratory methods

#### Thick and thin smears

Blood samples were collected using finger prick blood. Thick and thin smears were stained with Giemsa. Parasite density was determined by counting the number of asexual parasites per 200 white blood cells, and calculated per μL using the following formula: numbered parasites × 8,000/200 assuming a white blood cell count of 8,000 cells per μL. Absence of malaria parasite in 200 high power ocular fields of the thick film was considered as negative. The slide readers were kept blinded to the treatment allocated and day of visit.

### Haematological and biochemistry analysis

Haematological and biochemical tests were performed at enrolment and at Day 7 to evaluate haemoglobin concentration, creatinine, aspartate amino transferase (ASAT), alanine aminotransferase (ALAT), and bilirubin concentration. Biological abnormalities were noted.

### Genotyping

PCR analysis was done to distinguish recrudescence from new infection. Nested polymerase chain reaction (PCR) was conducted to compare two polymorphic genetic markers from *P. falciparum msp1* and *msp2* genes [12]. After DNA extraction, PCR was performed using primer pairs. Two microlitres of DNA in a total of 50 μL of reaction mixture were amplified during the primary PCR using the programme shown in Table 1. One microlitre of the product of the first amplification in a total of 50 μL of reaction mixture was used for the second PCR with the second programme. Each reaction mixture contained 10 μL of buffer/MgCl2 (3.5 mM), 5 μL of dNTP (200 mM), 0.5 μL of each primer (100 μM), 0.25 μL of Taq polymerase (0.5 units), and 32.25 μL of distilled DNA-free water. The DNA was added last. Amplification products were visualized by photography under ultraviolet light after electrophosis on 1.5% agarose containing ethidium bromide for *msp1* and 1.8% agarose for *msp2*.

**Table 1:** Primer sequence and polymerase chain reaction (PCR) program for MSP1 and MSP2 amplification

**Gene**	**Name**	**Primer sequence**	**PCR program**
msp 1 primary	O1	5^′^ CACATGAAAGTTATCAAGAACTTGTC 3^′^	94°C-3 min, [94°C-25 sec, 50 °C-45 sec, 68°C-2 min] × 30, 72°C-3 min
	O2	5′ GTACGTCTAATTCATTTGCACG 3^′^	
msp 1 nested	N1	5^′^ GCAGTATTGACAGGTTATGG 3^′^	94°C-3 min, [94°C-30 sec, 50 °C-45 sec, 68°C-2 min] × 30, 72°C-3 min
	N2	5^′^ GATTGAAAGGTATTTGAC 3^′^	
msp2 primary	S3	5^′^ GAAGGTAATTAAAACATTGTC 3^′^	94°C-3 min, [94°C-30 sec, 42′C-60 sec, 65°C-2 min] × 30, 72°C-3 min
	S2	5^′^ GAGGGATGTTGCTGCTCCACAG 3^′^	
msp2 nested	S1	5^′^ GAGTATAAGGAGAAGTATG 3^′^	94°C-3 min, [94°C-30 sec, 50 °C-60 sec, 72°C-2 min] × 45, 72°C-3 min
	S4	5^′^ CTAGAACCATGCATATGTCC 3^′^	

The difference in fragment size between pre-treatment and post-treatment samples allows a classification in three categories: new infection if the fragment sizes are different, recrudescence if the fragment sizes are similar, and both recrudescence and new infection if two fragments are visualized in the post-treatment sample with one similar to the pre-treatment fragment. The last two are considered as recrudescence for the analysis [13].

### Statistical methods

The number of subjects to be included in this study was calculated using STATA-11™ software, based on an expected therapeutic efficacy of AL at 98% [8] assuming a non-inferiority margin of 7% and 80% of power, using a 95% confidence level, sample size for each arm was evaluated at 160 patients. Data were entered in Excel™ software and analysed using STATA-11™ software. Characteristics of all patients included in the study were tabulated by study arm. For categorical data, percentage was used to assess the frequency of each outcome. For continuous data, mean and standard deviation were used to describe normally distributed variables; otherwise median and range were used. For the primary end point of adequate clinical and parasitological response at day 28, analysis was done by intention to treat and per protocol. The intention treat analysis included all randomized patients, while in the per protocol analysis, patients who were not seen at day 28 were excluded. The ACPR at day 28 was calculated for each study arm and compared using chi square test. The cumulative incidence of failure rate was calculated in each study arm and compared using Kaplan Meir method. For the ACPR at days 42 and 63, analysis was done by per protocol and comparison was made in a similar way.

The frequency of adverse events was described in terms of proportion in each study arm. For some adverse events such as dizziness, analysis was adjusted by haemoglobin level at inclusion, gender, age group and study arm. A stepwise logistic regression was done for the determination of factors associated with dizziness. Significance level of tests was set on 0.05 two side.

### Ethical considerations

The protocol was reviewed and approved by the Conseil National de Recherche en Santé (CNRS), which is the Senegalese national ethics committee. On the field, informed consent was requested from all participants prior to their enrolment.

## Results

### Baseline characteristics

**Figure 1:**
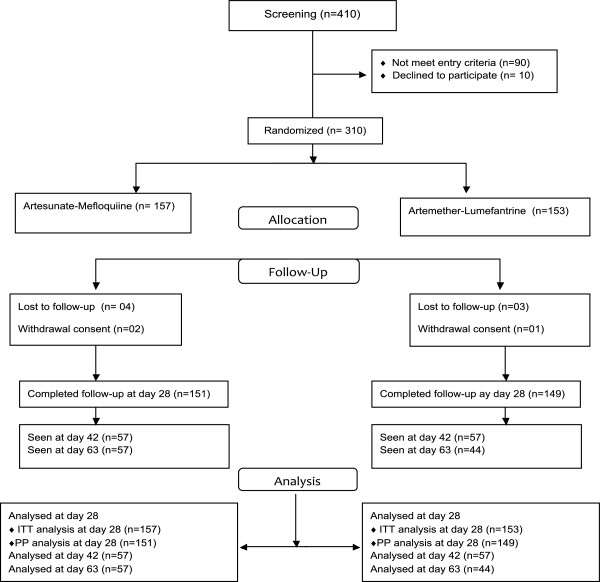
Patients’ trial profile.

Overall 410 individuals participated to the screening, and 310 of them meet the entry criteria. Patients’ trial profile is summarized in Figure 1. A total number of 310 patients, 157 in the AM arm and 153 in the AL arm were thus recruited in the study. At enrolment, patient characteristics were similar in the two study arms. No significant difference was noted in terms of age, axillary temperature, parasitaemia at inclusion, mean haemoglobin as well as biochemical parameters such as creatinine, ASAT, ALAT and bilirubin. Patient baseline characteristics are summarized in Table 2.

**Table 2:** Baseline characteristics of patients at enrolment in the two groups

	**Artesunate Mefloquine (n = 157)**	**Artemether Lumefantrine (n = 153)**	**p value**
Age (mean ± SD, years)	25 ± 11	23.2 ± 10,5	0.15
Weight (mean ± SD, Kg)	62.1 ± 11.8	58.2 ± 11.5	0.08
Sex ratio (male/female)	1.2	0.6	0.03
Temperature (mean ± SD)	37.8 ± 1.2	37.7 ± 1.1	0.41
Median Parasitemia (trophozoites/μL)	17411	17688	0.88
Mean haemoglobin (g/dl)	12.2 ± 1.7	11.7 ± 1.7	0.99
Anaemia (Hb < 11 g/dl,%)	36.3	40.5	0.44
ASAT (UI/L, mean ± SD)	31.2 ± 18.7	33.1 ± 16.8	0.38
Patients with normal level of ASAT (ASAT < 40 UI/L) (%)	128 (81.5%)	123 (80.4%)	0.79
ALAT (UI/L, mean ± SD)	28.2 ± 20.3	26.3 ± 24.1	0.47
Patients with normal level of ALAT (ALAT < 40 UI/L) (%)	119 (82.1%)	138 (90.2%)	0.04
Creatinine (mean ± SD)	7.6 ± 5.4	8.2 ± 3.5	0.24
Patients with normal level of creatinine (<13 mg/L) (%)	148 (94.3%)	143 (93.5%)	0.76
Median Bilirubunemia (mg/L)	1.91	1.71	0.51
Patients with normal level of Biliribunemia (<10 mg/L) (%)	48 (30.6%)	55 (35.9%)	0.31

### Treatment efficacy

**Figure 2:**
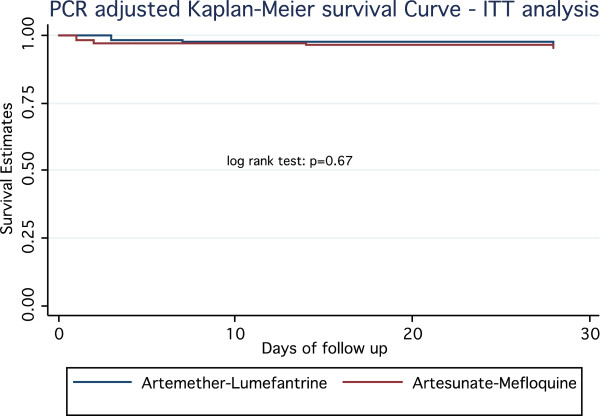
Kaplan Meir survival estimates of PCR adjusted ACPR by treatment arm – ITT analysis.

In the intention to treat analysis, PCR corrected adequate clinical parasitological response (ACPR) at day 28 (primary end point), was evaluated at 95.5% in the AM arm versus 96.7% in the AL arm (p = 0.58) (difference −0.01 (95%CI [−0.05;0.03]). In the per protocol analysis, the PCR adjusted ACPR was estimated at 99.33% in the AM arm versus 99.34% in the AL arm (p = 0.99) (difference 0.0008 95%CI [−0.018; 0.018]). In addition, the Kaplan-Meier PCR adjusted analysis did not show any significant difference between the two treatment groups in terms of cumulative incidence of failure rate, up to day 28 (log rank test, p = 0.67) (Figure 2). Thus, the non-inferiority of AM versus AL was demonstrated both in intention to treat and per protocol analysis (Table 3).

**Table 3:** Therapeutic efficacy of AM and AL at days 28, 42 and 63 in adult patients in Senegal – IIT and PP analysis

**Outcome**	**Artesunate Mefloquine (n = 157)**	**Artemether Lumefantrine (n = 153)**	**p value**
Intention to treat analysis			
Early treatment failure	00	00	-
NA	6 (3.82%)	4 (2.61%)	0.77
Crude Parasitological failure at day 28	1 (0.64%)	4 (2.61%)	0.35
PCR adjusted failure rate	1 (0.64%)	1 (0.65%)	
PCR adjusted cure rate at day 28	150 (95.54%)	148 (96.73%)	0.58
	**Artesunate Mefloquine**	**Artemether Lumefantrine**	
Per protocol analysis			
Early treatment failure	00	00	-
Crude parasitological failure	1/151 (0.66%)	4/149 (2.6%)	0.17
PCR adjusted failure rate	1/151 (0.66%)	1/149 (0.67%)	0.99
PCR adjusted cure rate at day 28	150/151 (99.34%)	148/149 (99.33%)	0.99
Crude parasitological failure at day 42	2/70 (2.8%)	5/57 (8.7%	0.20
PCR adjusted ACPR at day 42	69/70 (98.5%)	56/57 (98.2%)	1
Crude parasitological failure at day 63	4/57 (7%)	6/44 (13%)	0.32
PCR adjusted ACPR at day 63	56/57 (98.2%)	43/44 (97.7%)	1

NA = Not applicable.

For the 104 patients, representing 30% of the total sample, (57 AM arm and 57 AL arm) seen at day 42, the PCR adjusted ACPR was 98.5%% and 98.2%, respectively, in the AM and AL groups (Table 3). Overall, 57 patients in the AM arm and 44 patients in the AL arm attended the day 63 visit. PCR adjusted ACPR at day 63 was evaluated at 98.2%% in the AM arm versus 97.7% in the AL arm (p = 1) (Table 3).

The decrease of fever was similar in the two treatments groups. Following the first treatment dose (at day 0), 2.6% of patients in the AM arm were found with fever (temperature >37.5 °C) while 5.3% of patients in the AL arm were febrile after the first treatment day (p = 0.25). At day 2, all patients both in the AM arm and AL were found without fever.

The two treatments resulted in a rapid clearance of parasites. The mean parasitaemia at enrolment was 17,411 trophozoites/μL in the AM group and 17,688 in the AL group. It decreased to 864 trophozoites/μL in the AM group versus 949 trophozoites/μL in the AL group, 24 hours after the first dose (p = 0.28*).* Complete parasite clearance was obtained at Day 2 in the two treatment arms.

### Safety and tolerability

Overall, the two treatments were well tolerated. No serious adverse event was observed during the study period. The main observed adverse events, were dizziness, abdominal pain, vomiting, diarrhoea and pruritus (Table 4). Dizziness were more frequent in the AM arm (22.9%) than in the AL arm (11.1%) (p = 0.008). After controlling for confounding factors such as presence of anaemia at enrolment, age and gender, dizziness was closely associated with AM intake. Indeed, the adjusted odds ratio after the first dose of AM was 2.3 (95%CI 1.1-4.4; p = 0.02); an increased risk of dizziness was also found after the second dose of AM (aOR = 12.5; 95%CI 5.5-28.1; p = 0.001) and after the third dose (aOR = 10.2; 95%CI 4.7-22.3; p = 0.001) (Table 5).

**Table 4:** Incidence of adverse events in the two study groups from inclusion to day 7 of follow up

	**Day 1**		**Day 2**		**Day 3**		**Day 7**	
**Adverse event**	**AM**	**AL**	**AM**	**AL**	**AM**	**AL**	**AM**	**MF**
Dizziness (n,%)	35 (22.3)	17 (11.1)	61 (38.8)	8 (5.2)	58 (38.1)	11 (7.1)	15 (10.2)	6 (4.03)
Vomit (n,%)	16 (10.2)	11 (7.2)	09 (5.7)	0	04 (2.6)	0	0	0
Abdominal pain	15 (9.5)	11 (7.2)	11 (7.2)	10 (6.5)	10 (6.5)	4 (2.6)	1 (0.66)	0
Pruritus (n,%)	0	2 (1.3)	0	1 (0.6)	0	0	0	0
Oral herpes (n,%)	0	2 (1.3)	0	3 (1.9)	1 (0.66)	3 (1.9)	0	1 (0.67)

AM = Artesunate-mefloquine; AL = Artemether-Lumefantrine.

**Table 5:** Frequency of dizziness by study group adjusted by gender, age, anaemia at inclusion

**Dizziness**									
**Day 1**					**Day 2**			**Day 3**	
	**n (%)**	aOR** (95%CI)	**p value**	**n (%)**	**aOR (95%CI)**	**p value**	**n (%)**	**aOR (95%CI)**	**p value**
*Treatment									
**AL (n = 153)**	17 (11.1%)	1		8 (5.2)	1		11 (7.1)	1	
**AM (n = 157)**	35 (22.3%)	2.2 (1.1-4.4)	.02	61 (38.8)	12.5 (5.5 – 28.1)	.001	58 (38.1)	10.2 (4.7-22.3)	.001

* AM = Artesunate-mefloquine; AL = Artemether-Lumefantrine.

**aOR: adjusted odds ratio. The analysis of the association between dizziness and treatment was adjusted by mean haemoglobin at inclusion, age, gender.

Overall, patients’ biological profile was similar in the two treatments groups. Haemoglobin level was lower at day 7 in both AM and AL groups compared to haemoglobin level at inclusion in each study arm. Anaemia was more frequent at day 7 in the AL arm (68.6%) than in the AM arm (54.7%) (p = 0.01). An increased level of anaemia was observed in the two treatment groups at day 7 compared to the proportion of anaemic patients at enrolment. No significant difference was noted between the two treatment groups at day 7 in term of ALAT and ASAT level as well as bilirubin and creatinine concentration (Table 6).

**Table 6:** Patients’ biological profile at day 7 post treatment

	**AM**	**AL**	**p value**
	**Day 7**	**Day 7**	
Mean haemoglobin	11.3 ± 1.8	10.6 ± 1.5	0.11
Anaemia (%)	86 (54.7%)	105 (68.6%)	0.01
Median ALAT	19	19.3	0.93
Median ASAT	24	25	0.53
Patients with ASAT < 40 (%)	143 (91%)	139 (90.8%)	0.94
Patients with ALAT < 40 (%)	146 (92.9%)	145 (94.7%)	0.51
Mean creatinine	7.7 ± 4.7	8.2 ± 7.0	0.50
Patients with normal level of creatinine (%)	149 (94.9%)	150 (98.0%)	0.13
Median bilirubin	1.10	1.08	0.36
Patients with normal level of bilirubin (%)	92 (58.6%)	82 (53.6%)	0.37

## Discussion

Prompt treatment of malaria attacks with effective anti-malarial drug combination is an essential tool for malaria control. Malaria attacks treatment requires the use of artemisinin-based combination therapy (ACT). This non-inferiority randomized trial, evaluated the efficacy and safety of a new formulation of AM with 25 mg/kg mefloquine in comparison to AL. Results from this trial indicated that AM is as effective and well-tolerated as AL for the treatment of acute uncomplicated *P. falciparum* malaria in adults’ patients. Indeed, the PCR corrected ACPR at day 28 was 95.5% in the AM arm as against 96.7% in the AL arm; similar results were obtained in the per protocol analysis with a PCR corrected ACPR at 99.3% and 97.3%, respectively, in the AM and AL group.

These findings are consistent with data described in other trials conducted in African countries. Faye *et al.* in Senegal obtained a cure rate of 96.2% at day 28 when AM was administrated to children less than five years in a three-day treatment course [10]. Toure in Côte d’Ivoire reported a cure rate of 96% at day 28 for AM and 92% for AL [14], while Sowunmi et *al.* reported a therapeutic efficacy at 97% for AM and 94% for AL at day 28 in Nigerian children [15]. In patients seen at days 42 and 63 of follow up, PCR adjusted cure rate was evaluated at 98.5%% at day 42 and 98.2% at day 63 for AM group while that for the AL arm was at 98.2% at day 42 and 97.7% at day 63. Thus, the two treatments were highly effective at day 42 and 63 of follow up. Pharmacokinetics studies have demonstrated the long half-life of mefloquine and its ability to reduce rates of reinfection [16]. However, due to the low number of patients seen at day 42 and day 63, the study has lacked of power at day 42 and 62. Further studies with larger sample size would thus provide additional evidence on the long- term effect of mefloquine.

The two treatments were effective in reducing the parasite biomass as well as fever [5,6]. Thus, on day 2 a complete fever clearance was obtained in the two groups. Similarly, all study participants had a complete parasite clearance at day 2.

The two treatments were well tolerated with a similar safety profile. No serious adverse event was reported during the trial. However, dizziness was more frequent in patients treated with AM. The risk of dizziness in patients treated with AM was dose-related. Indeed, an increase risk was observed after each treatment course (aOR at day 1 = 2.3, aOR at day 2 = 12, aOR at day 3 = 10). Several reviews have published data on the neuropsychiatric effects of mefloquine. In more than 25,000 patients involved in about 20 trials, dizziness and anxiety were the most commonly reported adverse events in adult patients [17-19] as well as vomiting, especially in young children [20-22]. However, despite the frequency of dizziness during the study, no major biological disorder was noted in the study participants and therapeutic efficacy remains good in the two treatment groups. The use of the new formulation of AM should be combined with regular counselling in order to improve patient’s compliance.

## Conclusion

The new formulation of artesunate-mefloquine (25 mg/Kg mefloquine) is efficacious and well-tolerated for the treatment of uncomplicated *P. falciparum* malaria in adult patients.

## Competing interests

The authors declare that they have no competing interests.

## Authors’ contributions

RCT, BF, KS, JLN, OG conceived and designed the study. RCT, and KS monitored the data collection. RCT analysed the data. MN, ACL, AA were responsible for the PCR analysis. RCT wrote the first draft of the manuscript. All authors read and approved the final manuscript.
